# Dynamics of Vocalization-Induced Modulation of Auditory Cortical Activity at Mid-utterance

**DOI:** 10.1371/journal.pone.0060039

**Published:** 2013-03-28

**Authors:** Zhaocong Chen, Jeffery A. Jones, Peng Liu, Weifeng Li, Dongfeng Huang, Hanjun Liu

**Affiliations:** 1 Department of Rehabilitation Medicine, The First Affiliated Hospital, Sun Yat-sen University, Guangzhou, P. R. China; 2 Department of Psychology and Laurier Centre for Cognitive Neuroscience, Wilfrid Laurier University, Waterloo, Ontario, Canada; Hotchkiss Brain Institute, University of Calgary, Canada

## Abstract

**Background:**

Recent research has addressed the suppression of cortical sensory responses to altered auditory feedback that occurs at utterance onset regarding speech. However, there is reason to assume that the mechanisms underlying sensorimotor processing at mid-utterance are different than those involved in sensorimotor control at utterance onset. The present study attempted to examine the dynamics of event-related potentials (ERPs) to different acoustic versions of auditory feedback at mid-utterance.

**Methodology/Principal findings:**

Subjects produced a vowel sound while hearing their pitch-shifted voice (100 cents), a sum of their vocalization and pure tones, or a sum of their vocalization and white noise at mid-utterance via headphones. Subjects also passively listened to playback of what they heard during active vocalization. Cortical ERPs were recorded in response to different acoustic versions of feedback changes during both active vocalization and passive listening. The results showed that, relative to passive listening, active vocalization yielded enhanced P2 responses to the 100 cents pitch shifts, whereas suppression effects of P2 responses were observed when voice auditory feedback was distorted by pure tones or white noise.

**Conclusion/Significance:**

The present findings, for the first time, demonstrate a dynamic modulation of cortical activity as a function of the quality of acoustic feedback at mid-utterance, suggesting that auditory cortical responses can be enhanced or suppressed to distinguish self-produced speech from externally-produced sounds.

## Introduction

Forward models [1] are believed to play an important role in general motor control. These internal models use a copy of motor commands (i.e. efference copy) to predict the sensory consequences of one’s own action, and this prediction is compared with the actual outcome of that action. A match between the predicted and actual feedback results in a dampened sensory experience, while a mismatch results in an intensified sensory experience to allow the brain to allocate more attention to unexpected and important events from the environment [2]. The forward model has been successfully used to account for the interaction between motor and the visual system [3], somatosensory system [4]–[6], and auditory system [7]–[10].

As a highly skilled motor behavior, speech production involves the perception and monitoring of one’s own speech output. It has been suggested that the concept of the forward model can be also applied to speech production [11]–[13]. It has been well documented that activity in the auditory cortex is suppressed when the actual auditory feedback heard matches the feedback expected during vocal production. For example, several studies of single-unit activity in the auditory cortex of marmoset monkeys reported that self-produced vocalizations elicited suppressed neural discharges in the auditory cortical neurons [14]–[16], and that this suppression effect began several hundred milliseconds prior to the onset of vocalization [14]. Some magnetoencephalography (MEG) and functional magnetic resonance imaging (fMRI) studies in humans have also demonstrated that cortical responses to self-produced speech were significantly suppressed when compared with the activity observed while participants listened to playback of previously recorded self-produced speech [17]–[23]. In addition, several neurophysiological studies using electroencephalography (EEG) have identified a similar vocalization-induced suppression effect on the N1 component of the event-related potential (ERP) [24], [25]. And vocalization-induced suppression appears to be functionally related to the acoustic features of auditory feedback. For example, unaltered voice auditory feedback has been shown to elicit greater suppression of N1 responses compared with altered or alien auditory feedback [24], [25]. Moreover, this suppression was abolished when auditory feedback was completely masked by the white noise [17], [21].

It is noteworthy that suppressed responses to unaltered or altered auditory feedback reported in the above studies were evoked at the onset of vocal production. For example, Houde et al. [17] evaluated the MEG signal at the audio onset of each utterance, and Behroozmand et al. [24], [26] recorded the EEG signals to pitch shifts in auditory feedback triggered at utterance onset. According to the forward model, an efference copy is generated during motor planning and is used to produce a prediction of the auditory feedback that should be received by the auditory system. A mismatch between the predicted and received auditory feedback creates an error signal that modulates auditory cortical responses to incoming auditory feedback. At utterance onset, the efference copy enables the forward model to precisely predict auditory feedback. When the prediction closely matches the feedback received, only a small prediction error is generated and the auditory cortical responses are maximally suppressed. When listening to playback of self-produced vocalizations, however, motor planning does not occur so that the forward model does not generate a prediction, so responses in the auditory cortex are not suppressed. It has been suggested that the error signal that results from a mismatch between the forward model prediction and the actual sensory feedback enables the audio-vocal system to distinguish self-produced speech from externally-generated sounds [25], to correct for vocal errors during ongoing speech production, and to optimize the internal model for future productions [27]. Moreover, as the size of the difference between the expected and actual feedback increases, the prediction error becomes larger, resulting in the reduction of vocalization-induced suppression [20], [25].

Recently, several ERP studies have been conducted to explore the vocalization-induced auditory cortical activity at mid-utterance [24], [26], [28], [29]. In these studies, auditory feedback was unexpectedly pitch-shifted in the middle utterance of a vowel sound, and cortical responses to active vocalization and passive listening were recorded and compared. The results showed that, unlike previous studies of vocalization-induced suppression at utterance onset, active vocalization elicited larger cortical responses (P2) than passive listening, indicating a vocalization-induced enhancement effect at mid-utterance [26], [28], [30]. Moreover, the suppression effect was observed only when pitch shifts occurred at the vocal onset, while the enhancement effect was elicited only if pitch shifts were presented at a certain delay relative to the vocal onset [26]. These findings demonstrate that auditory cortical activity can be enhanced to detect the unexpected changes in auditory feedback at mid-utterance. And they provide evidence that neural mechanisms underlying the processing of auditory feedback are sensitive to the timing of delivery of auditory feedback alteration.

Vocalization-induced suppression at utterance onset has been successfully accounted for by the efference copy mechanism instantiated in the forward model [17], [25], [31]. Mechanisms underlying the vocalization-induced enhancement at mid-utterance, however, remain unclear. Behroozmand et al. [26] proposed that the enhancement effect induced by active vocalization at mid-utterance was primarily driven by the elimination of the suppression effect on the auditory neurons that existed at utterance onset. This explanation, however, is in contrast with the finding that vocalization-induced suppression at utterance onset persisted for the duration of self-produced vocalization in primates [14]. Moreover, although it has been demonstrated that suppression of early auditory activity (N1) at utterance onset is feedback specific [17], [26], it is not known whether the enhancement effect induced by active vocalization at mid-utterance is modulated as a function of the feedback quality or generalizes to any auditory signal heard after utterance onset. There is evidence that the mechanism involved in vocalization-induced enhancement may be less sensitive to the quality of the acoustic feedback than the mechanism involved in cortical suppression. For instance, it was found that enhancement occurred to mid-utterance pitch shifts as large as half an octave (500 cents) [28], while suppression did not occur for pitch shifts this large [24].

In the present study, we sought to examine the dynamics of vocalization-induced cortical responses to different acoustic versions of auditory feedback at mid-utterance. In the experiment, subjects sustained a vowel phonation while they heard their voice feedback either shifted in pitch (100 cents) or distorted by pure tones or white noise during active vocalization. Following the active vocalization condition, the recorded acoustic feedback signals were played back to the subjects during a passive listening condition. Cortical ERP (N1/P2) responses to feedback changes were obtained across conditions. We expected to see a feedback-specific cortical processing of auditory feedback at mid-utterance. That is, cortical responses induced by active vocalization relative to passive listening would be dynamically modulated by the acoustic features of auditory feedback.

## Methods

### Ethics Statement

All subjects signed the informed consent in compliance with a protocol approved by the Institution Review Board of The First Affiliated Hospital at Sun Yat-sen University of China.

### Subjects

Sixteen native Mandarin-speaking adults (8 women, aged 21–27 years) participated in this study. All subjects were right-handed, and they reported having no history of hearing, speech, or neurological disorders. All subjects passed a hearing screening test at the threshold of 25 dB HL for octaves from 500 to 4000 Hz for both ears.

### Experimental Design

**Figure 1:**
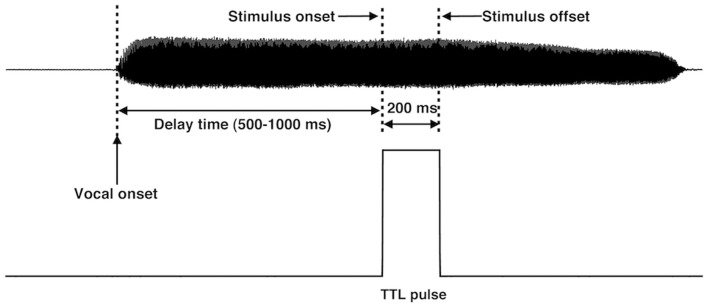
Schematic depicting the presentation of the acoustic stimulus in the middle of an utterance. After a random delay (500–1000 ms) with respect to the vocal onset (first dashed line), the acoustic stimulus was triggered (second dashed line) and lasted 200 ms (third dashed line). One TTL pulse was generated and sent to the recording system to mark the onset and offset of the acoustic stimulus.

The experiment consisted of three blocks of active vocalization and three blocks of passive listening. During active vocalization, subjects were instructed to sustained a vowel sound/u/for about 2–3 seconds. In one of the three blocks with active vocalization, the subjects heard their voice pitch-shifted upward 100 cents (100 cents equals one semitone) during each utterance. The duration of pitch shift stimuli (PSS) lasted for 200 ms. Unlike previous studies that the feedback alterations occurred at utterance onset [17], [24], [25], voice pitch feedback was altered 500–1000 ms after vocal onset in the present study (see Figure 1). A sum of voice auditory feedback and a sinusoidal tone (477 Hz, 200-ms duration, 5-ms onset and offset ramps, 80 dB SPL) or white noise (0–22 kHz bandwidth, 200-ms duration, 90 dB SPL) was presented to the subjects in the other two blocks. Subjects were asked to take a short break (2–3 seconds) between successive utterances and repeated the vocalization 80 times for each block, leading to a total of 240 trials for three blocks of active vocalization. Each active vocalization condition was followed by a passive listening condition, in which subjects listened to the playback of their self-produced vocalization. The order of three blocks of active vocalization was randomized across all subjects.

### Apparatus

Subjects were seated in a sound-treated booth throughout the experiment. Their vocal productions were recorded through a dynamic microphone (Genuine Shupu, model SM-306) and amplified with a MOTU Ultralite Mk3 firewire audio interface. In one condition, the amplified voice signals were pitch-shifted through an Eventide Eclipse Harmonizer. A custom-developed MIDI software program (Max/MSP v.5.0 by Cycling 74) was used to control the parameters of the pitch shifts (e.g., direction, duration, and magnitude) through the Eventide Eclipse Harmonizer. In the other two conditions, the MIDI program mixed the pure tones or white noise with the voice auditory feedback and fed back to the subjects. Subjects heard the altered auditory feedback through Etymotic earphones (model ER1-14A, Etymotic Research Inc.). The microphone and insert earphones were physically calibrated so that the intensity of feedback channel was 10 dB SPL higher than that of subject’s voice. This gain was used to partially mask air-born and bone-conducted voice feedback. Each subject’s voice onset automatically activated the MIDI program using a locally fabricated Schmitt trigger circuit that detected a positive voltage on the leading edge of the amplified vocal signals. The output of this circuit was used to trigger the pitch shifts, pure tones or white noise with a delay of 500–1000 ms with respect to the vocal onset.

After each block of active vocalization, the recorded feedback sound was played back to the subjects during the block of passive listening. The gain during passive listening with respect to active vocalization was carefully calibrated to ensure the audio level of the playback vocalization was the same as that of the self-produced vocalization [24], [26]. Two methods were employed for this calibration of the gain. One was the use of the sound level meter and a coupler to ensure that the intensity level of the sounds fed to the insert earphones during passive listening was identical to that during active vocalization. On the other hand, subjects were asked to verify that the amplitude of voice loudness during passive listening and active vocalization was nearly identical. The MIDI program generated the transistor-transistor logical (TTL) control pulses to indicate the onset and offset of each stimulus (see Figure 1). The voice, feedback, and TTL pulses were digitized at a sampling frequency of 10 kHz by Powerlab A/D converter (model ML880, AD Instruments) and recorded using LabChart software (v7.0 by AD Instruments).

### EEG Recording and Analysis

The EEG signal was recorded from the subject’s scalp using a 64-channel Geodesic Sensor Net and amplified with a Net Amps 300 (Electrical Geodesics Inc., Eugene, OR). The electro-oculogram (EOG) artifact was monitored with four electrodes placed above and below the eyes and at the outer canthus. Prior to the EEG recording, individual sensors were adjusted until impedances were less than 50 kΩ [32]. During the recording, all electrodes were referenced to the vertex (Cz) and the EEG signal was sampled with a frequency of 1000 Hz.

After data acquisition, the EEG signal was analyzed off-line using Net Station software (v.4.4, Electrical Geodesics Inc., Eugene, OR). All the channels were digitally bandpass-filtered from 1 to 20 Hz. The continuous EEG was segmented into epochs starting at 200 ms before and 500 ms after the stimulus onset. Segmented trials were then inspected for artifacts with the Artifact Detection toolbox in Net Station using a threshold of 50 µV for excessive muscular activity, eye blinks, and eye movements. Artifact-free segments were averaged, re-referenced to the average of electrodes on each mastoid and baseline corrected across all tasks. The amplitudes and latencies of the N1-P2 complex were extracted for statistical analyses, which were respectively measured as the negative and positive peaks in the time windows of 80–150 ms and 150–280 ms relative to the stimulus onset.

### Vocal Response Measurement

Event-related averaging techniques were used to measure the scale of vocal response to 100 cents PSS [33], [34]. In a custom-developed IGOR PRO (v.6.0, Wavemetrics Inc.) program, F_0_ values were calculated from the voice signals using the autocorrelation method in Praat [35] and then converted to cents scale using the formula: cents = 100×(39.86×log_10_(F_0_/reference)). The reference is frequency of an arbitrary note at 195.997 Hz (G4). The cents waveforms were segmented into epochs ranging from −200 (pre-stimulus period) to 700 ms relative to the onset of pitch perturbation. All segmented trials were waterfall displayed for the removal of bad trials prior to the averaging. One overall response was finally obtained by averaging the rest of the trials for each condition. Response magnitude was measured by subtracting the pre-stimulus mean from the peak value of the cents waveform.

### Statistical Analysis

Repeated-measures analyses of variance (RM-ANOVA) were conducted to examine effects of stimulus category (100 cents PSS, pure tones, white noise), task (vocalization, listening) and electrode site (FC3, FC1, FCz, FC2, FC4, C3, C1, Cz, C2, C4) on the amplitudes and latencies of N1 and P2 components. These electrode sites were chosen for statistical analyses because previous research showed that ERPs to pitch shifts at mid-utterance were primarily pronounced at the frontal-central electrodes [36]. Appropriate sub-RM-ANOVAs were calculated if higher-order interactions were observed. Probability values were corrected using Greenhouse-Geisser if the assumption of sphericity was violated. Corrected *p* values were reported along with original degrees of freedom.

## Results

**Figure 2:**
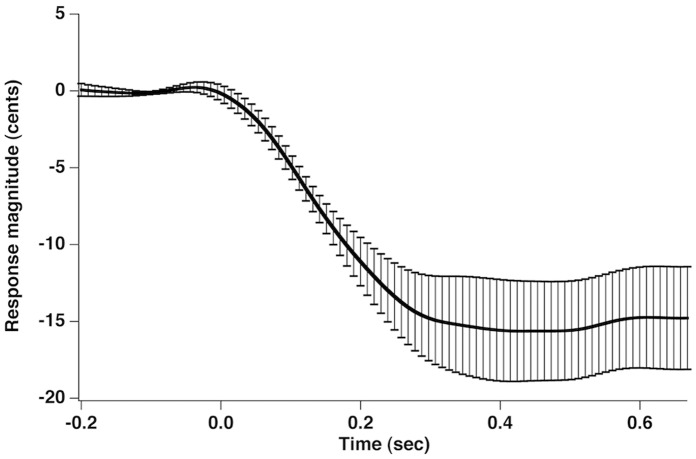
Grand-averaged voice F_0_ contours in response to 100 cents PSS. The vertical bars indicate the standard errors of averaged contours. The stimulus onset was at time 0.

**Figure 3:**
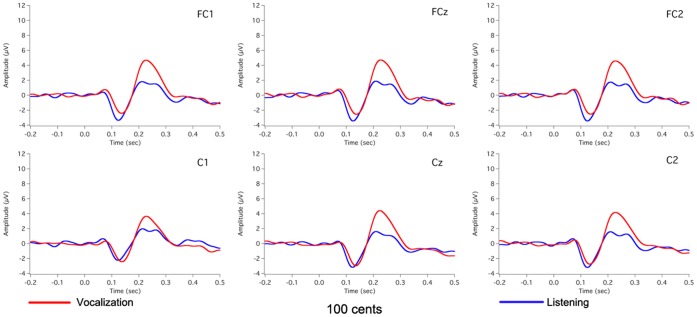
Grand-averaged waveforms of ERPs to 100 cents PSS during active vocalization (red traces) and passive listening (blue traces) at electrode sites of FC1, FCz, FC2, C1, Cz, and C2.

**Figure 4:**
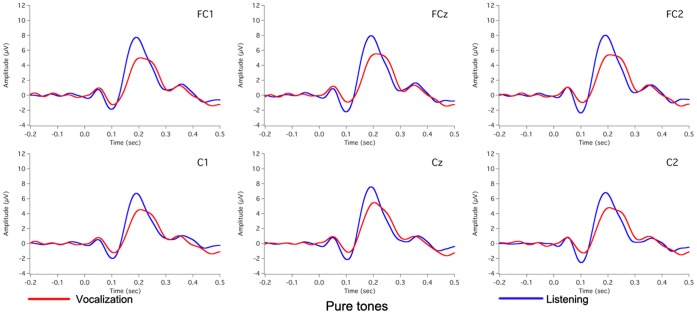
Grand-averaged waveforms of ERPs to pure tones during active vocalization (red traces) and passive listening (blue traces) at electrode sites of FC1, FCz, FC2, C1, Cz, and C2.

**Figure 5:**
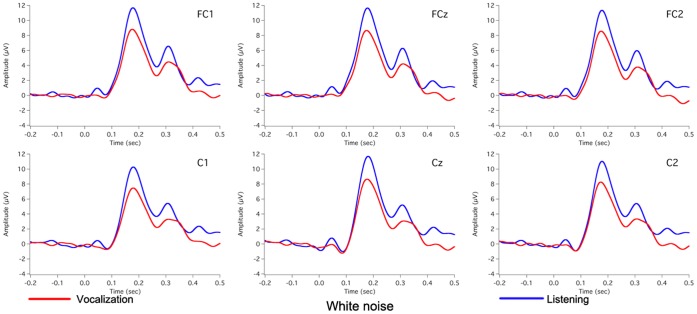
Grand-averaged waveforms of ERPs to white noise during active vocalization (red traces) and passive listening (blue traces) at electrode sites of FC1, FCz, FC2, C1, Cz, and C2.

**Figure 6:**
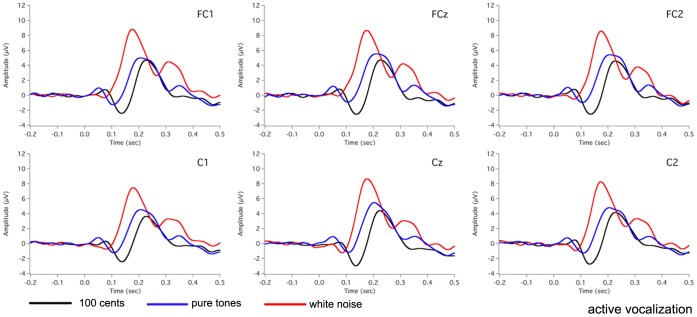
Grand-averaged waveforms of ERPs to 100 cents PSS (black traces), pure tones (blue traces), and white noise (red traces) during active vocalization at electrode sites of FC1, FCz, FC2, C1, Cz, and C2.

**Figure 7:**
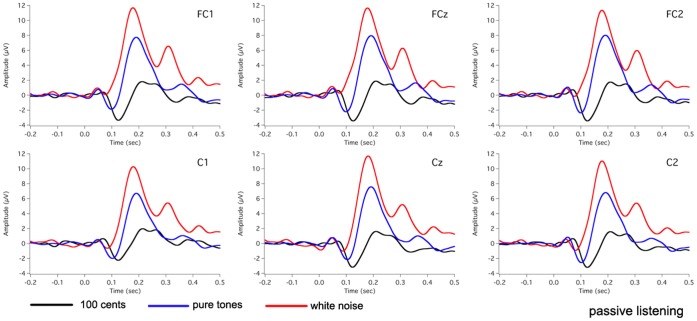
Grand-averaged waveforms of ERPs to 100 cents PSS (black traces), pure tones (blue traces), and white noise (red traces) during passive listening at electrode sites of FC1, FCz, FC2, C1, Cz, and C2.

Figure 2 shows the grand-averaged voice F_0_ contours in response to 100 cents PSS, in which vertical bars indicate the standard errors of averaged contours. All subjects produced compensatory vocal responses to upward 100 cents PSS by lowering their voice F_0_. The mean value of vocal responses to 100 cents PSS is 18 cents (SD: 12 cents). Figures 3, 4, 5 show the grand-averaged ERP waveforms during active vocalization (red traces) and passive listening (blue traces) for 100 cents PSS, pure tones, and white noise, respectively. As can be seen, active vocalization elicited larger P2 amplitudes than passive listening for 100 cents PSS. By contrast, P2 amplitudes for active vocalization were attenuated relative to passive listening for both pure tones and white noise. Figures 6, 7 show the grand-averaged ERP waveforms for 100 cents PSS (black traces), pure tones (blue traces), and white noise (red traces) during active vocalization and passive listening alone. Regardless of the experimental task (i.e. vocalization or listening), white noise elicited the greatest P2 amplitude, followed by pure tones and 100 cents PSS. And 100 cents PSS was associated with the longest P2 and N1 latencies compared with the other two stimuli. A series of RM-ANOVAs were performed on the amplitude and latency of P2 and N1 components across conditions and the results are described below.

### P2 Component

**Figure 8:**
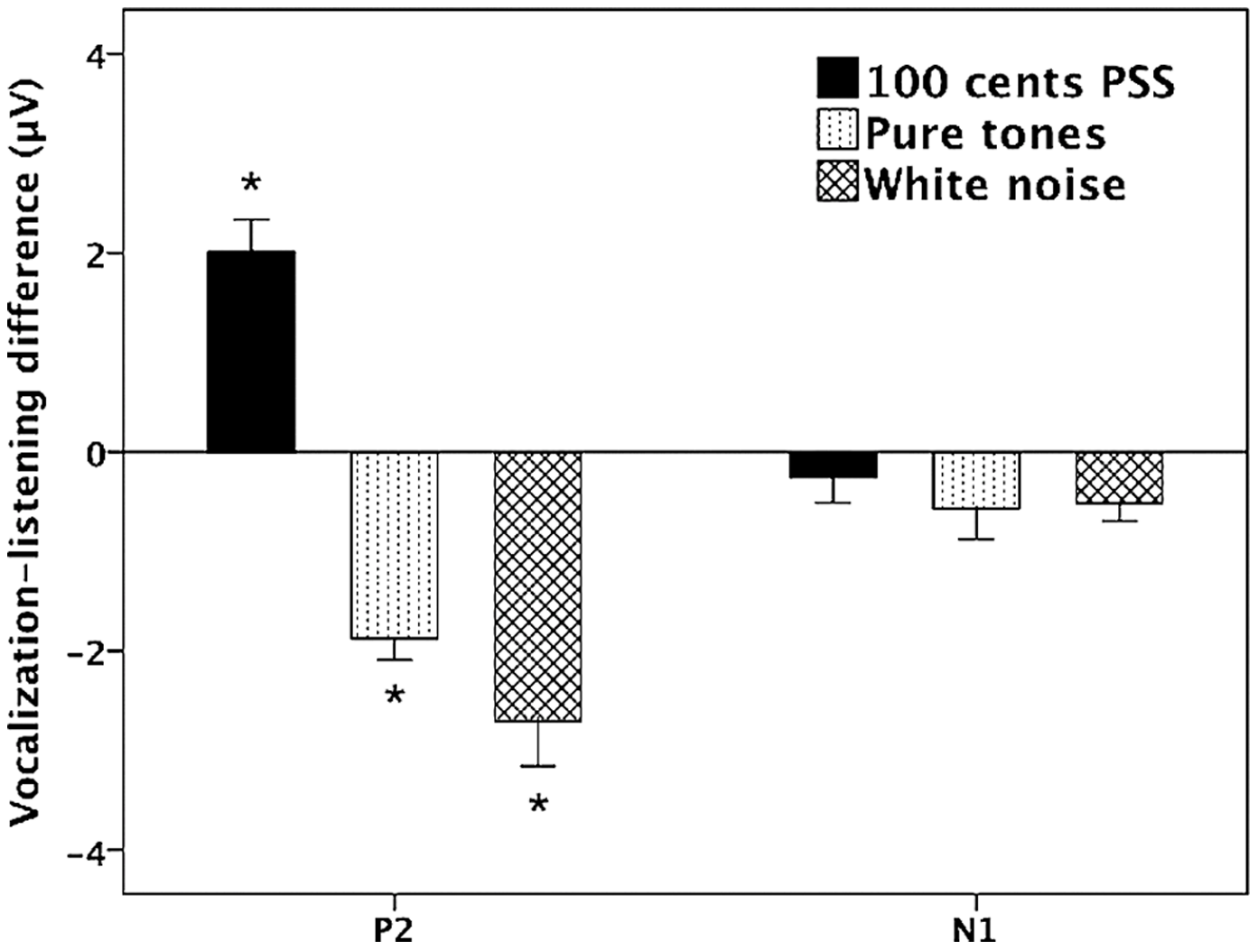
T-bar plots of the vocalization-listening difference (means and standard errors) of P2 and N1 amplitudes for 100 cents PSS, pure tones, and white noise. The positive and negative amplitudes of vocalization-listening difference denote vocalization-induced enhancement and suppression effect, respectively. The asterisks indicate significant differences of amplitude between active vocalization and passive listening.

**Figure 9:**
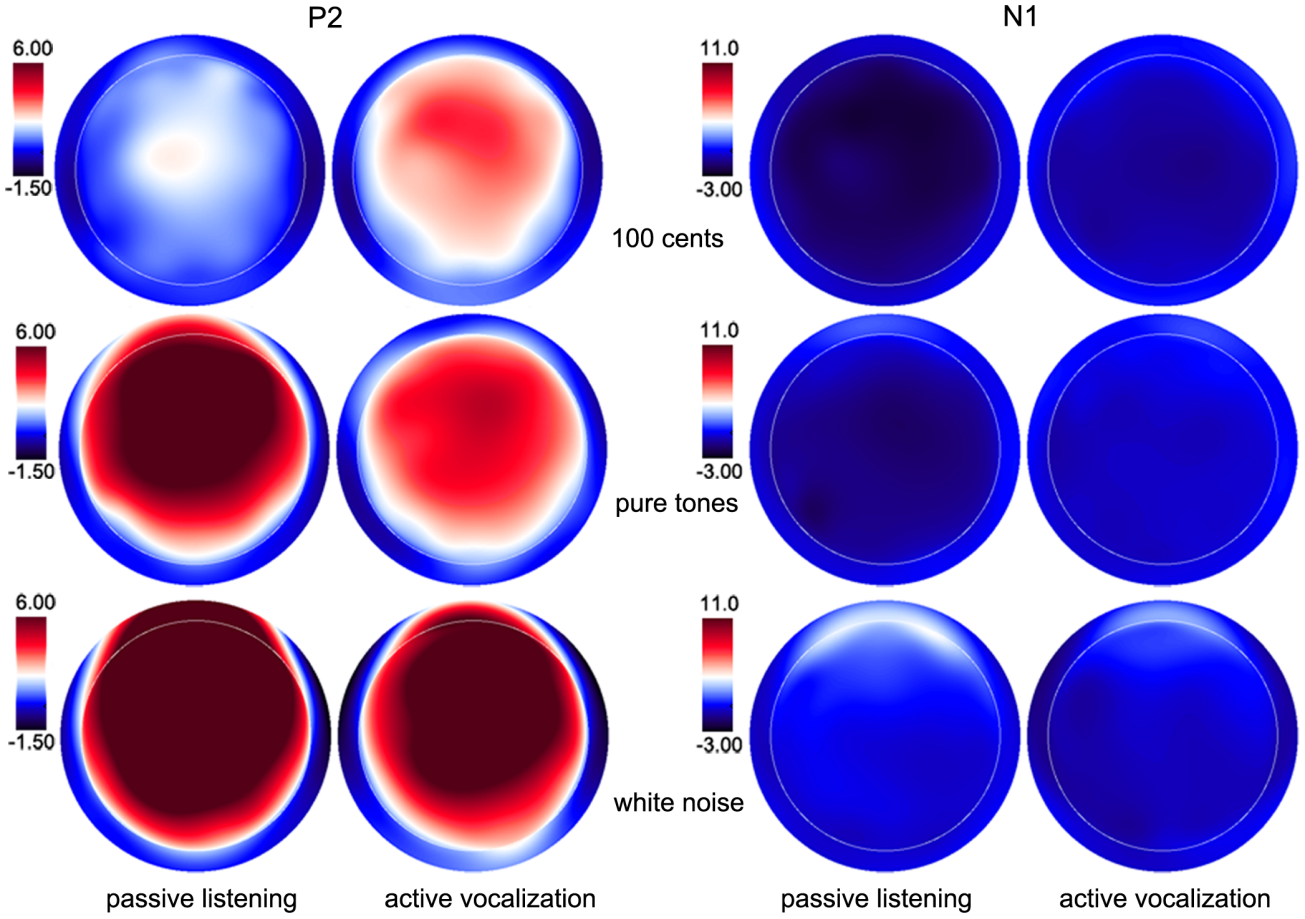
Topographical distributions of the grand-averaged ERPs during active vocalization and passive listening. From top to bottom are shown the respective ERP distributions for 100 cents PSS (top), pure tones (middle), and white noise (bottom). ERP distributions of P2 and N1 components are shown on the left and right column.

A three-way RM-ANOVA of P2 amplitude showed significant main effects of task (F(1, 15) = 6.667, p = 0.021), stimulus (F(2, 30) = 37.833, p<0.001) and site (F(9, 135) = 22.924, p<0.001). A significant interaction was found between task and stimulus (F(2, 45) = 28.255, p<0.001) led to separate task×site RM-ANOVAs for each stimulus. A significant main effect of task observed for the 100 cents PSS (F(1, 15) = 16.904, p = 0.001) revealed that P2 amplitudes were significantly larger for active vocalization compared with passive listening (see Figure 3). The main effect of task also reached significance for pure tones (F(1, 15) = 30.770, p<0.001) and white noise (F(1, 15) = 17.669, p = 0.001), but active vocalization elicited significantly smaller P2 amplitudes than passive listening (see Figures 4, 5). The T-bar plots in Figure 8 and topographical distributions of ERPs in Figure 9 show these enhancement or suppression effects for 100 cents PSS, pure tones, and white noise.

Separate stimulus×site RM-ANOVAs of P2 amplitude were also performed for active vocalization and passive listening, respectively. The results showed a significant main effect of stimulus during active vocalization (F(2, 30) = 13.579, p<0.001), and Bonferroni-adjusted comparisons revealed larger P2 amplitudes for white noise relative to 100 cents PSS (p = 0.001) and pure tones (p = 0.012) (see Figure 6). Similarly, there was a significant main effect of stimulus for the passive listening condition (F(2, 30) = 76.343, p<0.001), where significant differences were found between all the stimuli (p<0.002). The largest P2 amplitudes were associated with white noise, followed by pure tones and 100 cents PSS (see Figure 7).

In addition, statistical analyses of P2 latency revealed significant main effects of task (F(1, 15) = 6.774, p = 0.020) and stimulus (F(2, 430 = 48.298, p<0.001). Active vocalization elicited longer P2 latencies than passive listening (207±4 ms vs. 197±5 ms). White noise elicited the shortest P2 latency (178±3 ms), followed by pure tones (203±6 ms) and 100 cents PSS (225±5 ms) (see Figures 6, 7).

### N1 Component

For N1 amplitudes, one three-way RM-ANOVA showed significant main effects of stimulus (F(2, 30) = 6.984, p = 0.009), and site (F(9, 135) = 3.617, p = 0.009) but not task (F(1, 15) = 3.487, p = 0.082). Bonferroni-adjusted comparisons revealed that white noise was associated with smaller N1 amplitudes (absolute value) than 100 cents PSS (p = 0.025) and pure tones (p = 0.021) (see Figures 6, 7). A significant interaction was found between stimulus and site (F(18, 270) = 4.575, p = 0.001), and the following task×site RM-ANOVAs revealed significant main effects of site for 100 cents PSS (F(9, 135) = 4.230, p = 0.004) and white noise (F(9, 135) = 6.770, p<0.001).

The results of N1 latency revealed a significant main effect of stimulus (F(2, 30) = 46.282, p<0.001) and a significant site×stimulus interaction (F(18, 270) = 2.719, p = 0.024). Bonferroni-adjusted comparisons revealed the shortest N1 latency for white noise (81±4 ms), followed by pure tones (103±4 ms) and 100 cents PSS (124±4 ms) (see Figures 6, 7). Further task×site RM-ANOVAs across three stimuli showed a significant task effect only for pure tones (F(1, 15) = 5.783, p = 0.030), where passive listening elicited shorter N1 latencies than active vocalization.

## Discussion

The present study investigated the dynamics of auditory cortical activity to altered auditory feedback that occurred in the middle of an utterance during active vocalization and passive listening. As expected, active vocalization yielded enhanced P2 responses relative to passive listening when subjects heard the artificially produced pitch error (100 cents PSS). When voice auditory feedback was distorted by pure tones or white noise, however, a suppression effect was found as reflected by smaller P2 responses to active vocalization compared to passive listening. These findings demonstrate, for the first time, that enhanced and suppressed cortical processing of altered auditory feedback during mid-utterance, and they provide evidence that the auditory cortical activity observed in response to self-produced vocalization is not generally enhanced to all auditory signals but sensitive to the quality of the acoustic feedback.

In the present study, 100 cents PSS elicited enhanced P2 responses to active vocalization relative to passive listening, which is consistent with the results reported by Behroozmand et al. [28], [30]. Behroozmand et al. [28] also noted that the extent of enhancement (i.e., the amplitude difference between active vocalization and passive listening) decreased as the size of pitch shifts increased from 100 cents to 500 cents, suggesting that enhancement effect of cortical response to mid-utterance acoustic feedback varies as a function of the discrepancy between the predicted vs. actual feedback. The present findings further demonstrate that vocalization-induced response is not nonspecifically enhanced to all auditory signals at mid-utterance. Rather, the audio-vocal system dynamically modulates (i.e., enhances or suppresses) the cortical activity according to the nature of acoustic feedback.

With respect to pure tones and white noise, it is unexpected that active vocalization elicited attenuated P2 responses relative to passive listening. To the best of our knowledge, this is the first report of vocalization-induced suppression of P2 responses to alterations of auditory feedback that occurred at mid-utterance. Similar results were found in previous animal studies [14], [37], in which external acoustic stimuli (e.g. click trains, tones) presented at utterance onset resulted in attenuated responses compared with stimuli presented during passive listening. The present ERP finding complements the MEG results of humans reported by Houde et al. [17], in which M100 responses to pure tones were suppressed when subjects sustained vowel phonation compared with passively listening to both pure tones and tape-recorded vocalization. They also noted that the suppression effect was abolished when self-produced speech was distorted by gated white noise. Similarly, a recent fMRI study reported that the early activity in the auditory cortex to self-produced speech was no longer attenuated when speech feedback was completely masked by white noise [21]. By contrast, P2 responses to white noise induced by active vocalization were still suppressed relative to passive listening in the present study. Although specific explanations for these contrastive findings are not available, we speculate that neural mechanisms involved in the processing of auditory feedback at mid-utterance may differ from those at utterance onset. A further study that includes responses to feedback changes at both utterance onset and mid-utterance should be conducted to testify this speculation.

It might be argued that the inconsistence between the present study and previous research could be attributable to the language experience of the participants. Mandarin-native speakers were recruited in the present study, while English-native speakers were involved in most of previous research [17], [21], [25], [28]. Indeed, there is evidence that behavioral and neurophysiological responses to mid-utterance PSS are shaped by language experience [36], [38]. However, it is very unlikely that the vocalization-listening difference of ERPs would be specific to participants’ language experience. Several recent neurophysiological studies have demonstrated that cortical responses to mid-utterance PSS during active vocalization are enhanced relative to passive listening in either English or Mandarin participants [28], [29], [39], [40]. Therefore, the confounding factor of language experience would have not influenced on validity of our conclusions.

It is noteworthy that feedback changes presented at utterance onset in previous research were usually temporally predictable, while those occurred at mid-utterance in the present study were unpredictable. This confound leaves open a possibility that suppression or enhancement induced by vocalization observed in the present study may be related to the factor of temporal predictability. In a similar study that manipulating the timing of pitch shifts at mid-utterance as predictable or unpredictable [39], vocalization-induced suppression was found when the timing of pitch shifts was predictable, while enhancement effect was observed if subjects failed to predict their timing. This finding provides supportive evidence that suppression or enhancement of vocalization-induced responses to pitch shifts at mid-utterance is partly caused by the temporal predictability of feedback changes. This effect, however, cannot account for why vocalization-induced suppression effect was observed in the present study of white noise at mid-utterance but absent in other studies of white noise at utterance onset [17], [21]. If there were such an effect, a greater extent of suppression effect in response to white noise at utterance onset would have been observed because attenuated neural responses resulting from an accurate prediction of stimulus timing. Therefore, some other mechanisms should be responsible for the vocalization-induced suppression for white noise in the present study.

Findings from the present study and others [26], [30] have demonstrated vocalization-induced enhancement of cortical responses to mid-utterance pitch shifts. Behroozmand et al. [26] proposed that this enhancement effect resulted from the elimination of the masking effect of auditory cortical neurons suppression at utterance onset. Although not implausible, studies from single-unit recordings of the primate auditory cortex indicated that vocalization-induced suppression began several hundred milliseconds prior to vocal onset and persisted for the duration of self-produced vocalization [14]. One plausible explanation stems from the role of feedback in the online monitoring of self-produced vocalization. When the auditory feedback received mismatches the feedback predicted by a forward model, the speech motor control system registers the mismatch as a vocal error. Detecting this error is critical because it can be used to update the mapping between articulatory movements and their resultant vocal sounds to ensure that subsequent productions are accurate. So the sensitivity of the auditory system might be increased to detect these feedback errors, and the observed enhanced responsiveness to perturbations in auditory feedback may be related to this increased sensitivity. It has been reported in a recent single-unit recordings study on marmoset monkeys [16] that a majority of neurons (∼75%) in the auditory cortex exhibited increased firing rates during pitch-shifted feedback compared with the baseline condition (i.e., unaltered feedback). This type of intensified processing of feedback alteration in the auditory cortex, might account for the vocalization-induced enhancement effect for 100 cents PSS observed in the present study.

According to the above speculation, vocalization-induced enhancement effect can be generalized to any mid-utterance auditory signals. However, vocalization-induced suppression effect was observed in the present study when subject heard their voice distorted by pure tones or white noise. One possible explanation is that the audio-vocal system modulates its activity according to the quality of acoustic feedback. It has been demonstrated that sensory cortical activity can be modulated according to the feedback quality at utterance onset [17], [21], [24], [25]. A match between the predicted and unaltered auditory feedback resulted in the greatest suppression of auditory cortical activity induced by active vocalization [24], [25], and the suppression effect was less pronounced or even abolished with the decreasing of the feedback quality [17], [21], [24]. In an analogous way, exposing speakers to different versions of acoustic feedback may also result in a dynamic modulation of the auditory cortical activity at mid-utterance. Generally, a small perturbation to voice auditory feedback (e.g. 100 cents PSS) can be perceived as a natural fluctuation of one’s own voice. It has been suggested that the auditory-vocal system is optimally suited for stabilization of the voice around small perturbations [28], [41], [42]. Moreover, studies of vocal marmosets showed that their auditory cortex is sensitive to natural fluctuations of self-produced vocalization [15]. If this were the case, the sensitivity of the auditory cortex might be increased for the detection of those small pitch errors in order to update the current state of internal model of vocal production [43], which may be responsible for the enhanced cortical responses to 100 cents PSS during active vocalization in the present study.

By contrast, the quality of voice auditory feedback was seriously distorted by pure tones or white noise, perhaps causing it to be perceived as an external sound rather than a natural fluctuation of the speaker’s voice. According to control theory, the feedback-based control system attenuates the influence of sensory feedback when the feedback is delayed or distorted [44]. In particular, Houde and his colleagues proposed a state feedback control (SFC) model that involves Kalman filtering, which is used to convert the feedback prediction errors to state prediction errors that are used to refine vocal production [18], [43]. In this model, the gain of the Kalman filter on sensory feedback is proportional to the degree to which sensory feedback is uncorrelated with the current system. If the feedback is delayed or corrupted by other sounds such as noise, the Kalman filter largely attenuates the influence of feedback prediction errors on the correction of the current state estimate, resulting in small state prediction errors and the corresponding suppressed processing of sensory feedback. In the present study, therefore, the SFC model would convert a large feedback prediction error resulting from the distorted auditory feedback (i.e. pure tones or white noise) to a small state prediction error such that the vocal production can be properly controlled, leading to suppressed auditory cortical activity induced by active vocalization.

### Conclusion

The present ERP study investigated the dynamics of vocalization-induced auditory cortical activity at mid-utterance. The results revealed that, relative to passive listening, active vocalization elicited larger P2 responses when voice auditory feedback was pitch-shifted 100 cents. By contrast, attenuated P2 responses induced by active vocalization were observed when acoustic feedback was distorted by pure tones or white noise. These findings demonstrate the dynamics (e.g. enhancement or suppression) of auditory cortical activity in response to different acoustic versions of mid-utterance feedback alterations. It is suggested that the activity in the auditory cortex is not generally enhanced to all auditory signals but sensitive to the quality of the acoustic feedback at mid-utterance.
